# The Disordome Paradigm: How Intrinsic Disorder Encodes Emergent Biological Function

**DOI:** 10.3390/molecules31203662

**Published:** 2026-10-13

**Authors:** Jacques Fantini, Marine Lefebvre, Anaïs Aulas, Henri Chahinian, Nouara Yahi

**Affiliations:** 1 Unité de Recherche Microbes Evolution Phylogeny and Infections (MEPHI), IHU Méditerranée Infection, Aix-Marseille University (AMU), 13005 Marseille, France; 2 AmyPore, 13240 Septèmes-les-Vallons, France; marinelfv@gmail.com (M.L.);

**Keywords:** unfoldome, disordome, proteome, intrinsically disordered proteins, protein folding, electrostatic potential, quantum mechanisms, Alzheimer, Parkinson, gangliosides

## Abstract

Classical structural biology equates function with a fixed three-dimensional fold, a view challenged by intrinsically disordered proteins (IDPs) and regions (IDRs), which populate broad conformational ensembles rather than a unique structure. This article introduces the “disordome” paradigm, extending Uversky’s “unfoldome” to describe how function emerges from electrostatic and hydration landscapes that reshape reversibly upon encounter with a partner. Using α-synuclein binding to ganglioside-enriched rafts and IDR-driven liquid–liquid phase separation, we argue that transient folding-upon-binding events, potentially entropically cheap, generate information-rich complexes and condensates that dissolve once conditions change, consistent with the second law of thermodynamics. We trace this logic to overlooked precursors: in 1957 Klotz invoked rubber elasticity to introduce entropy as the driver of protein “disorientation”, while in 1962 Bernal described such proteins as chains “coiled in different ways” and formulated a “two-atom rule” whereby bonding constraints fix atomic geometry only up to two atoms apart. We argue that the local under-determination described by this two-atom rule, left unresolved in IDPs by any conserved network of contacts, helps explain why AlphaFold fails on IDPs. A quantum-mechanical pillar (dispersion, OH–π contacts, tunneling) is proposed as underrepresented in IDP research. We discuss implications for the origin of life, cellular regulation via condensates, and a therapeutic logic (“target the target, not the arrow”) illustrated by AmyP53, which occupies raft gangliosides rather than disordered amyloid proteins in Alzheimer’s and Parkinson’s diseases.

## 1. The Disordome: Transient Emergent Order in an Electrostatic Landscape

Classical structural biology has long been dominated by the paradigm “one sequence → one structure → one function“ [1,2]. In this view, structure is conceived as a well-defined, low-entropy three-dimensional arrangement of atoms, stabilized by a network of non-covalent interactions and wrapped in a shell of ordered water. Intrinsically disordered proteins (IDPs) and intrinsically disordered regions (IDRs) profoundly challenge this view [2,3,4]. They lack a single stable fold under physiological conditions, instead exploring a broad conformational ensemble [5,6,7,8,9,10]. Their “native” state is a dynamic cloud of possibilities rather than a unique structure [11].

**Figure 1:**
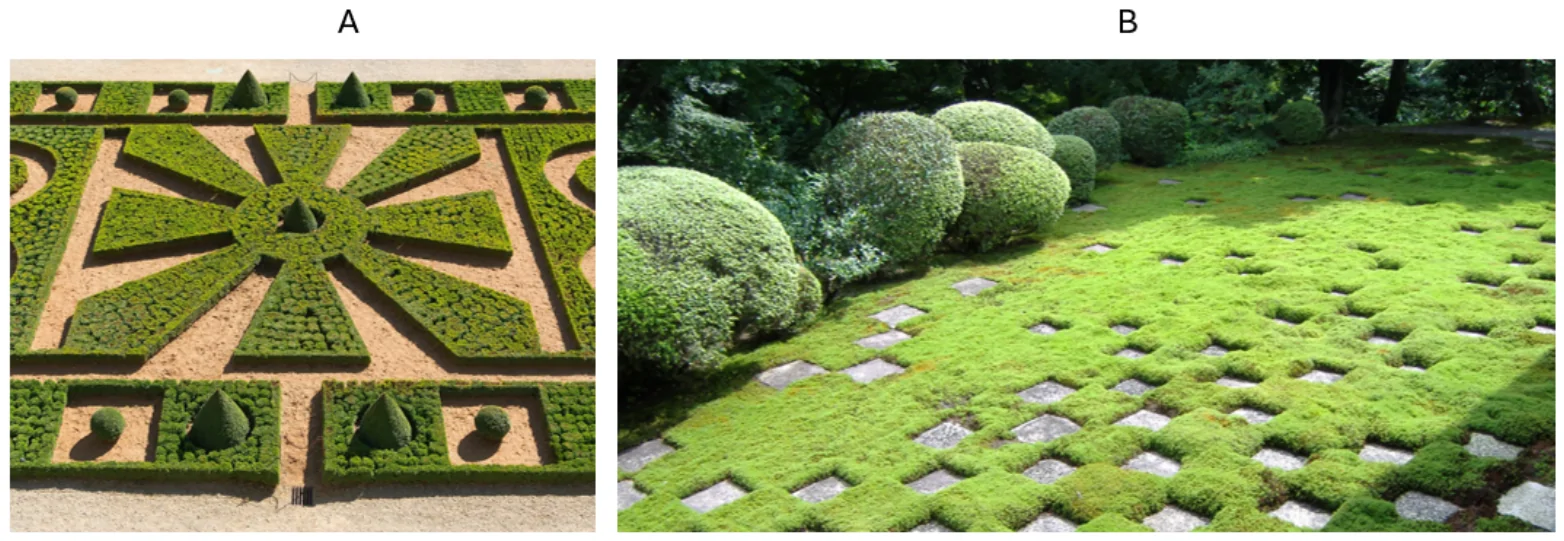
Two paradigms of order. (**A**) The French formal garden (Château de Hautefort) embodies the classical structure–function paradigm: a single, symmetric, geometrically fixed blueprint, invariant regardless of context. (**B**) The Japanese garden (Tōfuku-ji) embodies the disordome logic: no unique template constrains the arrangement of stones within the moss; multiple non-superimposable configurations achieve the same functional harmony, an aesthetic analogue of a degenerate conformational ensemble in which no single microstate is privileged. Credits: (**A**) by Jebulon, CCO https://commons.wikimedia.org/wiki/File:Ch%C3%A2teau_de_Hautefort_Jardins_3.jpg, (accessed on 10 September 2026) and (**B**) by Hiro2006, CC BY-SA 3.0 https://commons.wikimedia.org/wiki/File:Toufuku-ji_hojyo7.JPG (accessed on 10 September 2026).

This contrast between a single, obligatory architecture and a multiplicity of functionally equivalent configurations is not unique to molecular biology; it has a familiar visual analogue in two contrasting garden traditions (Figure 1). The French formal garden imposes a single, symmetric, geometrically fixed blueprint, reproduced identically regardless of context or observer: every parterre is a deterministic realization of one design (Figure 1A). The Japanese garden, in contrast, constrains its stones and moss by no such unique template: many non-superimposable arrangements of the same elements achieve an equivalent harmony, none of them structurally privileged over the others (Figure 1B). Classical structural biology has, until recently, modeled proteins almost exclusively after the first tradition, i.e., a genetically encoded blueprint (the amino-acid sequence) specifying one dominant three-dimensional fold, reproduced identically in every copy of the molecule. Intrinsically disordered proteins behave, instead, like the second: no single conformation is architecturally privileged, and yet, as this article will argue, functional coherence is nonetheless reliably achieved.

This raises a fundamental question: how can biological function emerge from such apparent structural indefiniteness? To address this, it is helpful to shift perspective from static structures to landscapes. At the atomic level, there is no “understanding” of what happens when an IDP engages a ligand or a partner protein. Each atom simply responds to the local electrostatic potential, which itself is continuously reshaped by the motions and mutual influences of all other charges and dipoles [12,13,14,15]. From this microscopic chaos, coherent patterns emerge at the molecular and supramolecular scale. In this sense, the disordered state of an IDP is not an absence of order, but a different mode of order: a distributed, latent order stored in the topology of its electrostatic landscape and in the statistics of its conformational ensemble. The protein exists in a “daily life” of disorder, constantly sampling many microstates. Some of these microstates are already pre-organized to be compatible with particular binding events. When a ligand or partner approaches, the long-range electrostatic field (screened at physiological ionic strength) steers the encounter, biases orientations, and selects among pre-existing conformations those that are “binding-competent”. Disorder is thus a temporal default, not a state of anticipation: no single folding outcome is inscribed in the sequence alone, since the eventual structure is co-shaped by whichever partner the protein happens to encounter.

The ensuing process often combines elements of conformational selection and induced fit: a suitable conformation is captured and then further refined as contacts accumulate.

**Figure 2:**
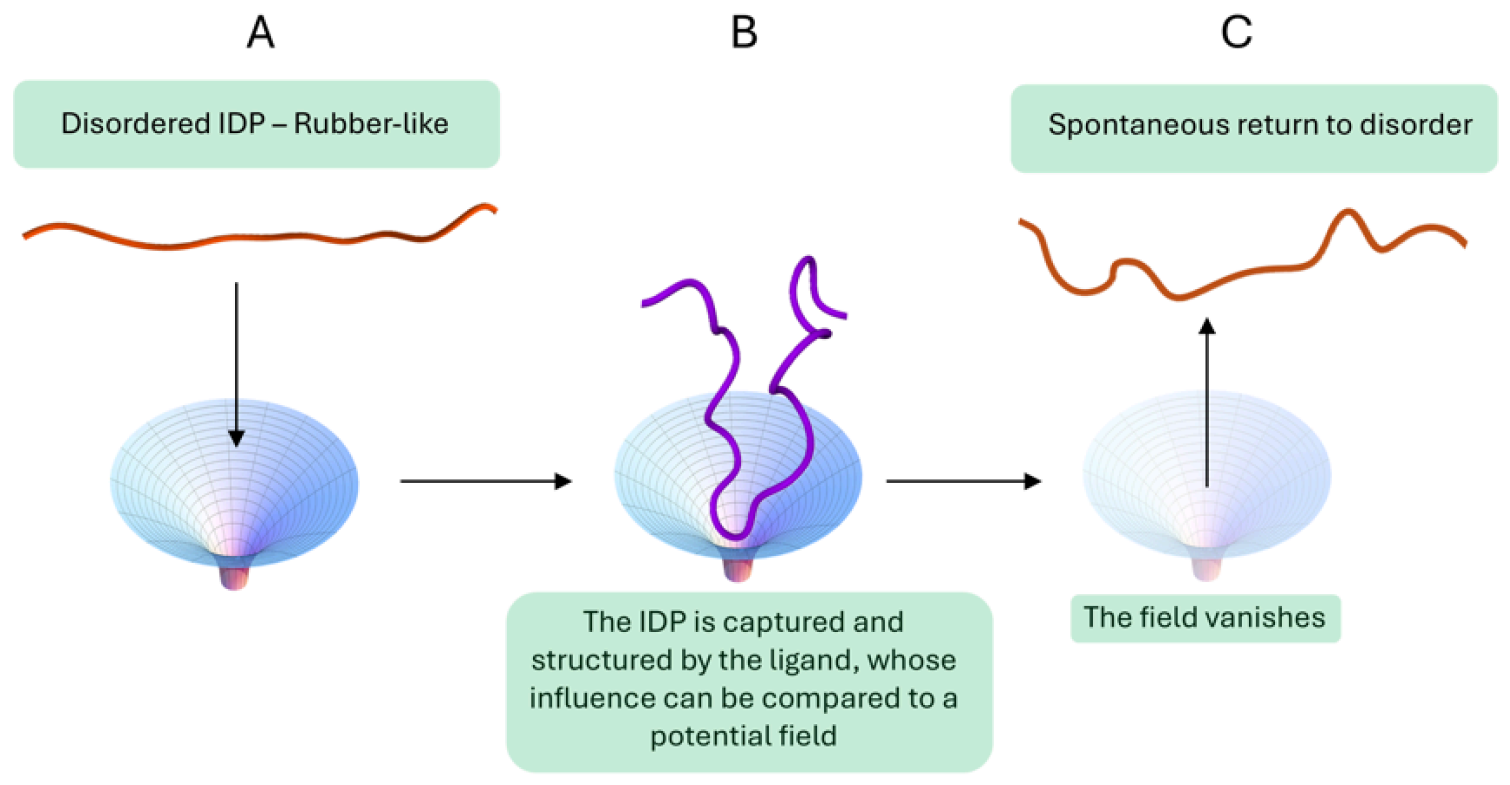
Electrostatic steering of an intrinsically disordered protein, avoiding a lock-and-key reading. This is a schematic representation of the conformational behavior of an intrinsically disordered protein (IDP) as it encounters a ligand. (**A**) In the absence of any partner, the free IDP is a rubber-like, fully disordered chain (orange), exploring a broad ensemble of conformations with no persistent secondary structure. (**B**) The ligand’s influence is represented as an electrostatic potential field (funnel), screened at physiological ionic strength, which locally reshapes the probability landscape experienced by the chain. Under this influence, the IDP is captured and transiently structured not because it physically docks into a pre-formed cavity, but because the field biases its conformational search toward a subset of pre-existing, binding-competent microstates. (**C**) Once the interaction ends and the field vanishes, the chain relaxes spontaneously back to the same equilibrium ensemble of disordered conformations it started from. The specific configuration shown here is a different microstate from the initial one, within the same ensemble. Reversibility thus applies to the statistical ensemble as a whole, not to any single, individually sampled microstate, which is not revisited identically along a stochastic trajectory.

A useful analogy is that of rubber [16,17] (see Historical Precursors, below). The polymer chain (Figure 2A) possesses an intrinsic capacity for deformation, encoded in its architecture, but no specific shape is imposed in the absence of an external constraint. Rather than picturing a rigid object docking into a complementary cavity (a lock-and-key reading that intrinsic disorder specifically defies) [18], the influence of a ligand is better represented as a long-range electrostatic potential field that locally reshapes the probability landscape experienced by the chain (Figure 2B). Under this influence, the chain is not physically molded around a solid shape, but captured and biased toward a subset of its own pre-existing, binding-competent microstates, which become transiently structured. Once the interaction ends and the field vanishes, the system relaxes spontaneously back to the same equilibrium ensemble, although a single chain occupies a different microstate (Figure 2C). In a similar way, an IDP can transiently acquire an ordered conformation upon binding, then relax back to its equilibrium disordered ensemble once the complex dissociates. The “shape” is thus an emergent, reversible, and statistically biased event and definitely not the imprint of a rigid mold, and not a permanent attribute.

This leads naturally to the distinction between the “unfoldome” and what may be called the “disordome”. Uversky coined the term “unfoldome” [11] to denote the genetic set of all intrinsically disordered proteins and regions within a proteome, emphasizing their lack of stable tertiary structure, a notion rooted in the idea of deviation from an underlying structural reference, since “unfolded” is defined only in relation to a folded norm. Here we propose the term “disordome” to designate the ensemble of intrinsically disordered proteins and regions whose functional behavior is not a departure from a folded norm, but a consequence of living natively on rugged, shallow energy landscapes; landscapes of which ganglioside-rich rafts are one particularly well-characterized instance [19], but which also encompass multivalent electrostatic interaction hubs [20,21] and the mesoscale, lipid-independent environments of biomolecular condensates [22]. In the disordome, function emerges from controlled, context-dependent collapses of highly flexible chains into transiently ordered states, orchestrated by electrostatic steering and multivalent interactions, whether these operate at a membrane surface, within a fuzzy protein complex, or inside a phase-separated droplet. The disordome is therefore not merely a catalog of disordered sequences [11], but the dynamic repertoire of context-imposed conformations and binding modes through which intrinsic disorder is harnessed to generate entropy-driven, specific recognition events, whether at the cell surface or elsewhere in a living system (see Defining the Disordome, below, for a full formalization).

Thermodynamically, these events can be viewed as local entropic emergences [23,24] within the framework of coupled folding and binding [25]. Complex formation reduces the conformational entropy of the IDP and of the ligand [26,27,28] and reorganizes the surrounding solvent, as shown for the p27Kip1–Cdk2/cyclin A interaction, where binding releases and reorganizes interfacial water [29]. Two features may soften this cost. First, the free ensemble can already include partially pre-organized states that spread out the entropic cost of ordering [30,31,32]. Second, many IDPs carry less tightly bound hydration water than globular proteins, which moves in and out more freely [33,34,35], so part of the entropic and enthalpic reorganization may fall on the ligand and its hydration rather than on the IDP itself. This behavior is, however, sequence- and context-dependent: water behaves as a good solvent for some IDPs and disordered segments, but not for all. Net charge, charge patterning and ionic strength determine whether water acts as a good, near-theta or poor solvent for a given disordered chain, with highly charged sequences tending to expand and low-charge polar sequences tending to collapse [36,37,38,39]. In this heuristic sense, IDPs may be “entropically cheap”, a hypothesis that remains to be formally quantified. After dissociation, the chain reverts to its equilibrium ensemble, as rubber recovers its shape without external energy input [40,41]: the return to disorder expresses the statistical dominance of the high-entropy state, as confirmed for model IDPs by entropic-elasticity measurements [42].

The concept of an “electrostatic landscape” [43] is central here. IDPs have taught us that rather than thinking of proteins as rigid objects in space, one can think of them as dynamically reshaping charge distributions embedded in a polar medium. The mutual influence of biomolecular partners on this landscape through their charge patterns, dipoles, polarizabilities, and spatial arrangements governs the probabilities of encounter [44], the efficiency of recognition [45], and the nature of the transient structures that appear. At each step, atoms only “see” local fields, yet at the supramolecular scale, recognizable functional forms emerge: complexes, scaffolds, and condensates [13].

This naturally extends to collective phenomena such as liquid–liquid phase separation and the formation of biomolecular condensates [46,47]. Many condensates are driven by multivalent, weak interactions between IDRs, often enriched in charged and aromatic residues [48,49]. Here, the emergent entity is not a folded domain but a mesoscale phase, a dense droplet whose physical properties (viscosity, internal dynamics, exchange with the dilute phase) are again governed by the organization of the electrostatic and interaction landscape across many chains [50,51]. Each individual chain or residue only responds to local forces, yet the ensemble produces a new level of organization, with its own characteristic timescales and functional roles in the cell [52,53].

All of these emergent structures, including folded complexes, transient helices, fuzzy interfaces, and condensates, are local decreases in entropy, organized in space and time [24]. They are not exceptions to the second law of thermodynamics, but manifestations of it, consistent with the general physical tendency of driving many-body systems to self-organize into dissipative, ordered states [54]. The cell continuously draws on free-energy sources and maintains fluxes (of metabolites, ions, electrons) [55] that allow these local entropic emergences to appear, perform work, store and process information, and then dissolve [56,57]. At a deeper conceptual level, life can thus be described as a process based on molecular interactions that organize controlled local entropic emergences, regulated by the mutual influences that biomolecular partners exert on the electrostatic landscape. These mechanisms, emergent by nature and “invisible” at the level of individual atoms, remain entirely compatible with the second law of thermodynamics, which governs the global increase in entropy at the scale of the universe [58].

In this framework, the disordome is not merely a catalogue of non-folded proteins; it is the arena where this logic is most clearly expressed. Intrinsic disorder makes visible what is actually true of all biology: function is not solely encoded in static three-dimensional structures, but in the capacity of matter to support, in a noisy and fluctuating environment, reproducible patterns of emergent order. Such patterns are sculpted by electrostatics, filtered by thermodynamics, and harnessed by evolution to create information-bearing, adaptive systems we call life.

## 2. A Concrete Illustration: Progressive Structuring of α-Synuclein on a GM3 Raft

α-Synuclein is a prototypical intrinsically disordered protein, found both in the cytosol and in the extracellular milieu [59,60], yet it can acquire an α-helical structure upon binding to lipid raft membrane microdomains enriched in gangliosides, a process thought to contribute to Parkinson’s disease [61,62,63]. In aqueous solution, the monomeric protein explores a broad ensemble of disordered conformations; no single three-dimensional fold dominates [64], and its hydration shell is highly dynamic, with water molecules continuously entering and leaving without forming a rigid, long-lived “bound” layer [34].

When a GM3-containing raft comes into play, the story changes. The charged and polar headgroup of GM3, including its sialic acid and carbohydrate moieties, begins to reshape the electrostatic landscape experienced by α-synuclein [19]. Individual residues do not “know” that a raft is nearby; each simply responds to the local electrostatic potential, while that potential is itself being modulated by the fluctuating positions and orientations of both the protein and the lipids. In this first stage, the interaction is essentially an electrostatically steered encounter: the protein’s conformational ensemble is biased toward orientations and microstates that favor approach and initial contact with the GM3 headgroups.

As α-synuclein draws closer, a subset of conformations within its ensemble becomes progressively more populated: those in which segments of the N-terminal region adopt amphipathic α-helical motifs capable of lying along the membrane surface. At the same time, water molecules that previously occupied the interfacial region between protein and membrane begin to redistribute. Some are expelled from between hydrophobic side chains and the lipid acyl chains, contributing a favorable gain in solvent entropy; others rearrange around newly formed charge–charge and hydrogen-bonding interactions between helical α-synuclein and the sialic acid/carbohydrate groups of GM3. The raft is not a passive template, but an active participant that co-sculpts the electrostatic and hydration landscape.

Step by step, this process gives rise to an emergent ganglioside-binding domain (GBD) on the protein surface [19]. No such domain exists as a stable structural entity in the free, disordered state; it is carved out dynamically as α-synuclein settles into a membrane-bound conformation in which the GBD engages GM3 with high specificity.

Consistent with a conformational-selection mechanism, high-resolution NMR studies show that the free monomer already retains a detectable, transient propensity for a helical structure within its broader N-terminal region [65], consistent with the first step of membrane engagement, in which this amphipathic segment progressively stabilizes into a continuous α-helix upon contact with the bilayer or micelle surface. This partial, transient resemblance between the free-state and membrane-associated conformations has since been confirmed and refined by subsequent studies [66].

This pre-existing propensity, however, does not extend to the GBD itself (residues 34–45) [67], whose characteristic turn conformation has no counterpart in the free-state ensemble and is not pre-organized prior to engagement. Rather, this turn is induced de novo by the specific, short-range OH–π contact between Tyr39 and the ganglioside glycone [68], once the protein has already been brought into proximity by the broader, conformational-selection-driven helical engagement. In this case, conformational selection and induced fit operate sequentially and cooperatively within the same binding event, rather than as competing explanations: a partially pre-organized helical propensity facilitates initial approach and membrane engagement, while the GBD’s distinctive turn, absent from the free ensemble, is subsequently sculpted by direct, partner-specific contact.

From the atomistic point of view, nothing magical happens: each atom continues to respond to its local environment, water molecules continue their picosecond dance, and lipids fluctuate within the bilayer. Yet, at the mesoscopic level, a recognizably structured, functional motif emerges: one that is capable of mediating raft localization, modulating synaptic function, and under pathological conditions, influencing membrane-assisted oligomerization pathways [69,70].

Crucially, as also illustrated in Figure 2, this structuring is both progressive and reversible. As long as the membrane interaction is maintained, the GBD persists as a locally ordered reduction in conformational entropy [67], stabilized by a balance of electrostatic, hydrophobic and hydrogen-bonding interactions, including the transient OH–π bond between Tyr39 and the ganglioside glycone, as suggested by molecular dynamics simulations [68] and probably facilitated by the reorganization of interfacial water. The GBD is thus not a permanent structural module in the classical sense, but a recurring event in the disordome, i.e., a transient shaping of the electrostatic and hydration landscape that appears, performs a function, and then dissolves.

**Figure 3:**
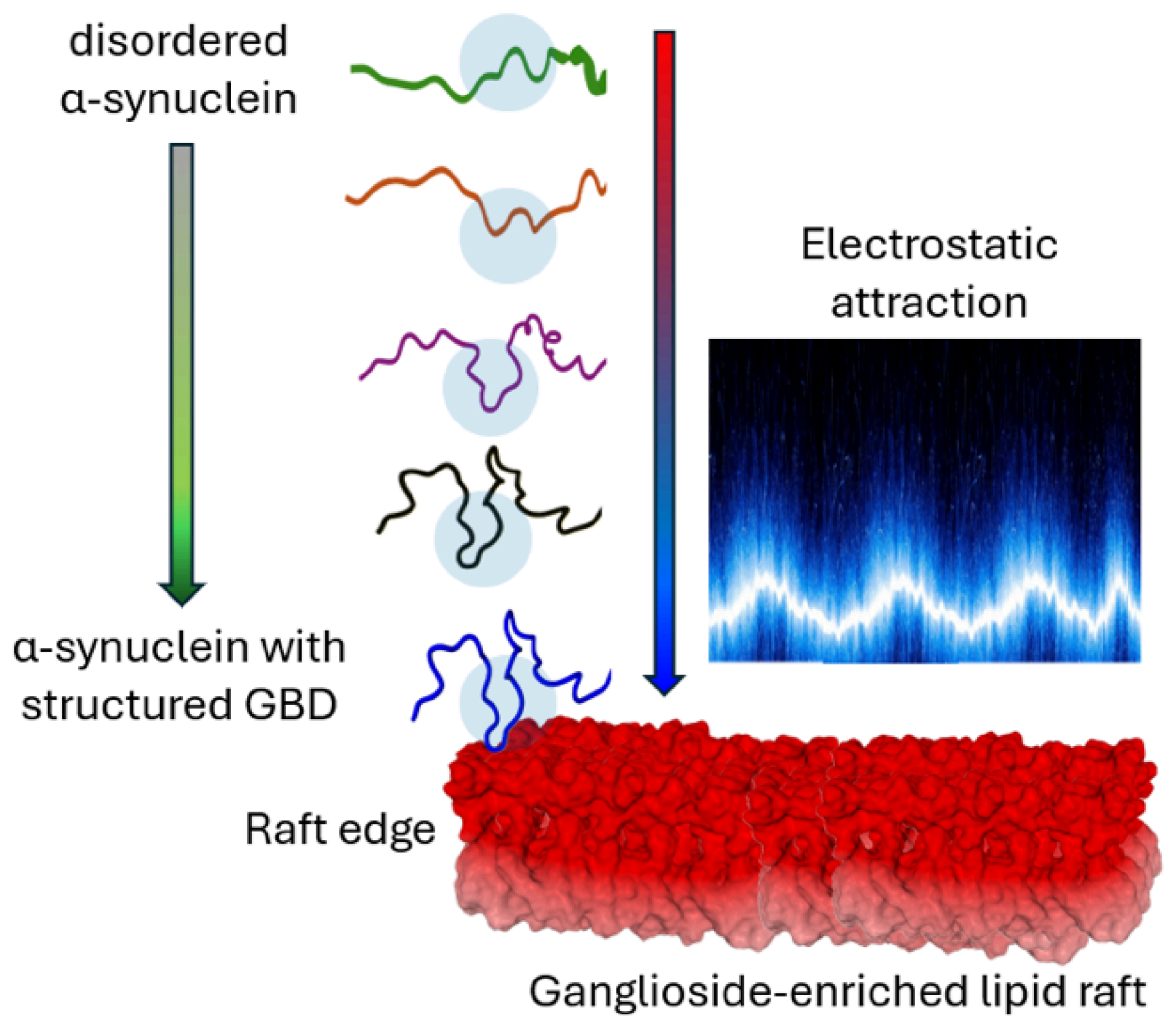
Electrostatically steered structuring of the α-synuclein ganglioside-binding domain during approach to a GM3-enriched raft. Schematic successive snapshots (top to bottom) of monomeric α-synuclein as it approaches the edge of a ganglioside GM3-enriched lipid raft. In the free, disordered state (top, green), the protein explores a broad ensemble of random-coil conformations, with no persistent secondary structure anywhere along the chain. As the protein approaches within approximately the Debye length of the raft periphery, electrostatic screening at physiological ionic strength limits the range of influence of the negatively charged, sialic-acid-bearing GM3 headgroups to roughly one nanometer, yet it can be locally extended by headgroup charge clustering (right; the blue-to-red gradient and the oscillating waveform are schematic devices illustrating the concept of a strengthening interaction with decreasing distance). Within this range, the central segment highlighted in light blue (i.e., the ganglioside-binding domain, GBD) progressively departs from randomness and adopts an increasingly compact, defined turn, consistent with 10 ns molecular dynamics simulations of the Tyr39/GM3 OH–π interaction previously reported by our group [68], while the flanking regions remain disordered throughout. By the time the protein reaches the raft edge (bottom, dark blue), the GBD has acquired a conformation favorably positioned for engagement with a GM3 dimer at the raft boundary [70]. We propose, as a working hypothesis, that this reflects progressive biasing of the ensemble toward a binding-competent state rather than rigid, pre-formed complementarity, with full multivalent, short-range contacts establishing specificity only upon direct engagement. The simulations describe the insertion phase only; any structuring before contact remains to be tested. If confirmed, this sequence would be a concrete molecular instance of the principle illustrated in Figure 2: structure is not a fixed attribute of the free protein but arises step by step as the disordered ensemble is biased by the electrostatic field of its partner.

In this example, the disordome logic is fully explicit. Going beyond the generic potential well of Figure 2, in which the target field is treated as fixed and one-sided, the GM3 raft edge examined in Figure 3 is itself a flexible, laterally mobile assembly. Peripheral gangliosides are not rigidly anchored: they can reorient and cluster into a dimeric, chalice-like conformation upon the approach of a charged ligand, a structural plasticity now documented for raft-edge gangliosides more generally. The resulting α-synuclein–GM3 complex therefore does exhibit an excellent structural fit between the folded ganglioside-binding domain and the GM3 dimer, but this fit is not the signature of a pre-existing lock-and-key complementarity between two static partners. It is the emergent outcome of a mutual induced-fit process, in which both the disordered protein and the fluid raft edge actively reshape each other’s conformational landscape until a jointly stable, low-energy configuration is reached (a mechanism already described for other intrinsically disordered complexes, such as Bim engaging its cryptic site on Bcl-xL [71]). Figure 2 thus captures the minimal, one-sided version of the disordome logic; Figure 3 reveals its full, reciprocal form, in which “landscape” and “ligand” are, at the raft edge, no longer cleanly separable categories.

This reciprocity carries a decisive therapeutic corollary. If the final, well-fitted α-synuclein–GM3 complex requires not one but two coupled conformational searches, the protein exploring its ensemble for a binding-competent turn, and the raft edge simultaneously reorganizing into its chalice-like dimer, then the overall recognition process is inherently slow, stochastic, and doubly vulnerable to kinetic interception. This is precisely the vulnerability exploited by AmyP53, an adaptive therapeutic peptide designed by combination of the amino acids constituting the GBDs of α-synuclein and Alzheimer’s β-amyloid protein (Aβ) [72]. As a short peptide with a reduced conformational search space, AmyP53 does not need to await this two-body co-adaptation: it collapses onto the raft surface and into its ganglioside-binding site, essentially instantaneously, occupying the landscape before either the protein’s or the raft’s slower conformational search can reach completion [19]. AmyP53 therefore does not out-compete α-synuclein or Aβ by binding “better” in a lock-and-key sense; it out-competes them in time, winning a race that intrinsic disorder, on both sides of the interaction, has made unavoidably slow. This mechanism of action has been characterized in various in vitro, ex vivo, and in vivo models [73]. Specifically, the kinetic advantage of AmyP53 is supported by three lines of evidence from our laboratory [69,74]: (i) in silico simulations of the competition between AmyP53 and amyloid proteins (either α-synuclein or Aβ) for ganglioside binding; (ii) the initial rate of insertion into a ganglioside monolayer, measured as the real-time increase in surface pressure, which is faster for AmyP53 than for α-synuclein; and (iii) the therapeutic effect of AmyP53, which prevents amyloid pore formation and calcium entry when competing with the amyloid protein for the membrane. Preclinical regulatory studies, including some performed independently by contract research organizations, support the tolerability of AmyP53 in the species and durations tested [69,75]. Repeated intranasal administration for 28 days in rats, and 14 days in rabbits, followed by a recovery period, at doses up to 5 mg/kg/day, caused no local or systemic toxicity, with a no-observed-adverse-effect level at the highest dose tested. In this respect, an important issue warrants discussion. Unlike current anti-amyloid immunotherapies, which rely on antibodies that recognize several forms of Aβ, including physiological monomers, AmyP53 is directed not at the protein, but at the pool of gangliosides engaged in the initiation of the neurotoxic cascade under pathological conditions. The physiological functions of monomeric amyloid proteins are therefore expected to be preserved, and preclinical studies indicate that AmyP53 is well-tolerated. Targeting gangliosides nonetheless raises legitimate safety questions, which we address below. Indeed, gangliosides regulate several membrane receptors by modulating their conformation through chaperone-like effects within lipid rafts [70]. As discussed by Krengel and Bousquet [76], the glycan headgroups of these regulatory gangliosides are physically associated with the receptors and are therefore masked, so that they are not accessible in trans to extracellular ligands. On this basis, interference of AmyP53 with regulatory gangliosides of membrane receptors appears unlikely [73].

Interestingly, the mutual induced-fit mechanism proposed here for the α-synuclein-GM3 complex is consistent with recent advances in computational protein design. RoseTTAFold (RF) diffusion-based binder design for IDPs and IDRs explicitly relies on “freely sampling both target and binding protein conformations”, with the target and the designed binder co-folding into a jointly stable, mutually adapted complex [77]. This is consistent with, though it does not by itself confirm, the idea that an excellent structural fit with a disordered protein does not require lock-and-key complementarity but can emerge from bilateral conformational adaptation, i.e., the same logic we attribute to the raft edge itself. It is worth stressing, however, that binder design for a disordered target and blind prediction of that same target’s own conformational ensemble are distinct tasks with distinct success criteria: these RFdiffusion binders are not evaluated against, and do not resolve, the ensemble-prediction problem that CASP16 shows remains open [78].

Yet, this convergence of mechanism must not be mistaken for a convergence of therapeutic philosophy. Baker-lab binders are designed to engage the disordered protein directly, e.g., G3BP1, the IL-2 receptor γ-chain, or amylin [77,79], all moonlighting proteins that perform essential cellular functions [80,81,82] that high-affinity sequestration risks disrupting. The disordome paradigm developed here suggests a different target for this same design logic: not the IDP itself, but the landscape, namely the membrane microdomain that recruits and structures it. Mutual induced-fit is a validated physical mechanism for engaging disordered systems [30]; where that engagement should be aimed is a separate, and consequential, choice. Target the target, not the arrow [19].

An important refinement is needed here. Work from our group has shown that amyloidogenic proteins that oligomerize into neurotoxic amyloid pores possess not one but two distinct, tandem lipid-recognition domains: the ganglioside-binding domain (GBD) already discussed, and a separate cholesterol-binding domain (CBD), located elsewhere in the sequence (residues 67–78 in α-synuclein, residues 22–35 in Alzheimer’s Aβ peptide) [83,84]. These two domains operate sequentially and have fundamentally different thermodynamic signatures. As long as the interaction is restricted to the GBD engaging a raft ganglioside, the protein-raft association remains what we have described above: a transient, reversible shaping of the electrostatic and hydration landscape, readily dissolved when the protein diffuses away from the raft edge. However, once the CBD inserts into the membrane and engages cholesterol, the picture changes qualitatively: this second step drives α-helical folding, a tilted membrane topology, and oligomerization into a calcium-permeable amyloid pore, and it is, for practical purposes, thermodynamically irreversible [85]. Consistent with this two-step model, the CBD alone (exemplified by the Aβ22–35 peptide) is sufficient to insert into cholesterol-rich membranes and self-assemble into neurotoxic amyloid pores, whereas the GBD alone is innocuous and cannot nucleate pore formation [85]. This asymmetry is not a minor structural detail: it is the rationale behind the design of AmyP53, a chimeric peptide built exclusively from the GBDs of Aβ and α-synuclein, deliberately excluding both proteins’ CBDs. By competing for the ganglioside-binding step without ever engaging cholesterol, AmyP53 blocks the initiating, reversible event of the amyloid cascade while carrying none of the pore-forming capacity intrinsic to the CBD [68].

## 3. A Second Illustration: Emergent Order in an IDR-Driven Condensate

**Figure 4:**
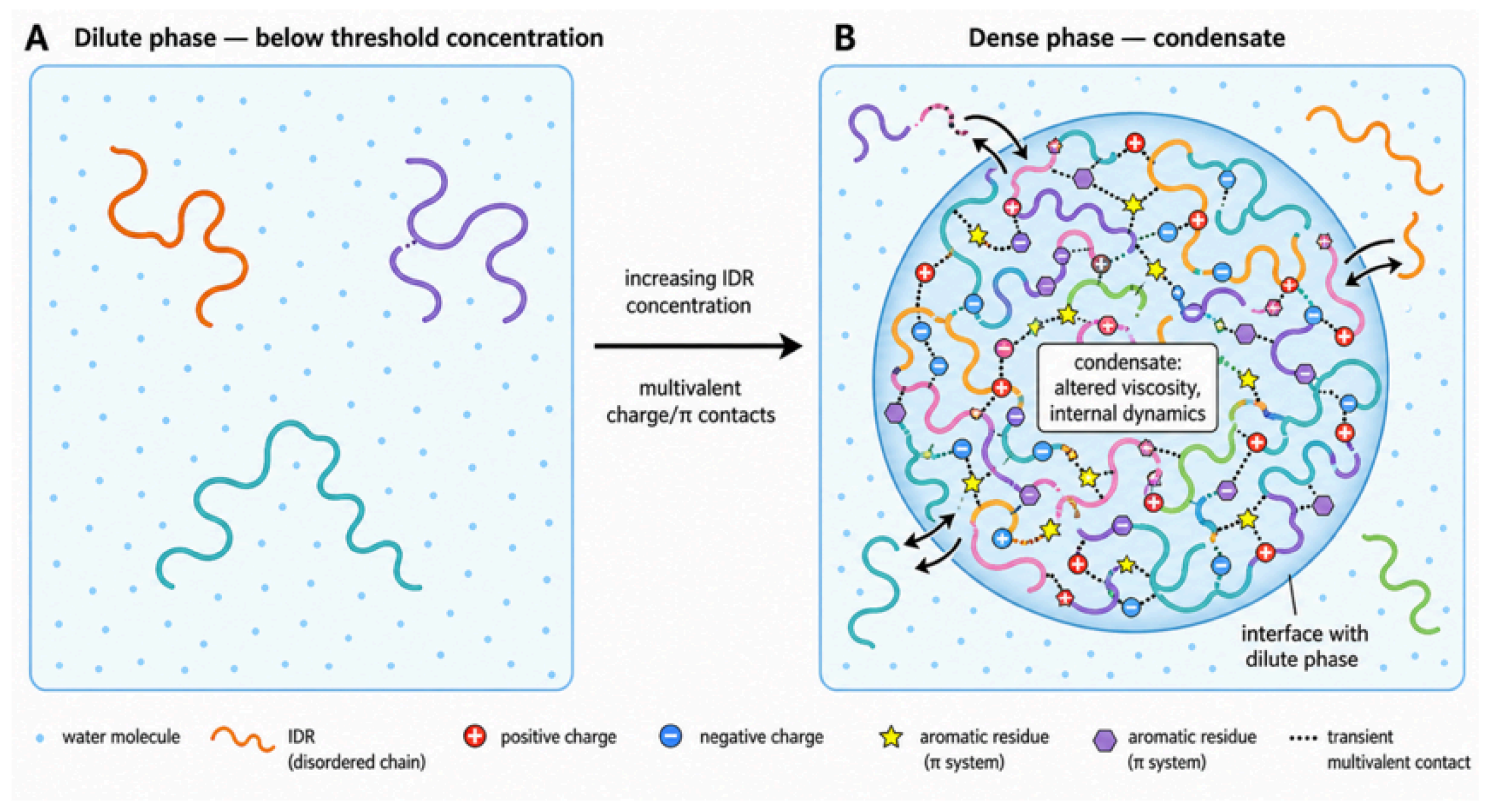
Liquid–liquid phase separation (LLPS) of an intrinsically disordered region (IDR). (**A**) In the dilute regime, below the critical concentration, individual IDR chains (shown in different colors) are dispersed throughout the aqueous solvent, each exploring a broad, independent conformational ensemble. Electrostatic interactions between residues are present but screened and fluctuating, and no persistent inter-chain contacts form; water molecules move freely around each chain without organizing into a rigid hydration shell. (**B**) As local IDR concentration rises above this threshold, multivalent contacts between charged (+/–) and aromatic (π-interacting) residues on different chains become increasingly frequent, nucleating a dense, protein-rich phase that coexists with the surrounding dilute phase: a biomolecular condensate. Within the droplet, individual chains remain dynamic and disordered at the single-molecule level, yet the assembly as a whole displays emergent collective properties: altered diffusion, increased local concentration of specific clients, and modified internal viscosity. The droplet boundary is not a rigid membrane but a dynamic interface, continuously exchanging chains with the dilute phase (curved arrows). This transition illustrates a second, collective mode of the disordome logic: order does not require any single chain to fold into a fixed structure, but emerges instead from the cooperative, reversible organization of many disordered chains within a shared electrostatic and hydration landscape.

Liquid–liquid phase separation (LLPS) of intrinsically disordered protein regions (IDRs) [20,22,48] provides a second, more collective, illustration of the disordome logic (Figure 4). Consider a charged low-complexity IDR that, at low concentration, remains fully miscible in the cytoplasm or nucleoplasm. In this dilute regime, the chain explores a broad conformational ensemble [86], and its hydration shell remains as dynamic as previously described for the free monomeric protein [33]. Electrostatic interactions between residues are present but screened and fluctuating; long-range correlations are weak [43].

As the local concentration of this IDR increases beyond a critical threshold [48], the balance of interactions changes. Multivalent contacts between oppositely charged or π-interacting residues on different chains become more frequent [87], and their cumulative effect reshapes the electrostatic and interaction landscape. Each individual residue continues to respond only to its local environment, but the local environment now increasingly reflects the presence of many neighboring chains. Water plays a dual role: it mediates transient interactions between polar and charged side chains, and it is partially excluded from regions where chains come into closer contact, gaining entropy as it is released into the bulk [88].

At a certain point, a new mesoscopic entity emerges: a dense protein-rich phase coexisting with a dilute phase, i.e., a condensate. Inside this droplet, IDRs are still dynamic and disordered on the level of individual chains [89], but the system as a whole exhibits new collective properties: altered diffusion coefficients [50], increased effective concentration of specific clients [22], and a modified pattern of secondary-structure propensities and backbone–backbone interactions [89]. The condensate has an interface with the surrounding solvent, an internal viscosity, and characteristic relaxation times [90], all of which depend on sequence features such as charge patterning, aromatic content [91], and post-translational modifications [92]. None of these properties can be inferred from a single chain in isolation; they arise from the interplay of many chains within a shared electrostatic and hydration landscape.

From the standpoint of the disordome, an IDR-driven condensate is thus not merely a “clump” of disordered proteins, but a higher-order entropic emergence. The transition from a homogeneous solution to a two-phase system involves local reductions in configurational entropy of the chains and local restructuring of water, compensated by favorable interaction enthalpies and an overall increase in the entropy of the entire system (proteins plus solvent plus surrounding environment). Within the droplet, secondary structures may form transiently, backbones may establish new interaction patterns [89], and specific clients may be selectively enriched or excluded [22], creating an information-rich microenvironment that modulates biochemical reactions [93].

Importantly, this emergent order is again reversible. Changes in salt concentration or temperature [87], post-translational modification [92], or partner abundance [22] can dissolve the condensate, returning IDRs to the dilute, highly hydrated, dynamically disordered state [46]. The condensate, like the ganglioside-binding domain of α-synuclein prior to cholesterol engagement by the adjacent CBD, is a transient event in the disordome: a context-dependent shaping of the electrostatic and hydration landscape that appears under appropriate conditions, performs regulatory and organizational functions, and then vanishes when conditions change.

In this way, LLPS makes explicit the central idea of the disordome: intrinsic disorder does not merely tolerate, but actively enables, the emergence of higher-order structures and phases whose stability, dynamics, and functional specificity are encoded in patterns of charges and interaction motifs, mediated by water, and constrained by thermodynamics.

This second illustration is gaining increasing recognition: a growing catalogue of RNA-binding proteins [94] and transcriptional regulators [95] exemplifies this behavior in living cells (Table 1).

A striking illustration that has grown in interest for the last few years is the assembly of stress granules (SGs). Triggered by cellular stress, these transient cytoplasmic aggregates sequester untranslated mRNAs and translation factors, increasing their local concentration by over tenfold relative to the rest of the cytoplasm [96,97]. In 2020, Yang et al. demonstrated that to assemble, stress granules need a network of 36 proteins that are either IDPs or contain IDRs [98]. G3BP1 is a tunable switch that triggers phase separation to assemble stress granules [98]. Overall, the SG proteome is enriched in IDP/IDR protein compared to the cytoplasm; each of them contributes to the reversible SG assembly by acting either as a node that expands the network through protein interactions or as a cap that terminates the growth of the assembly [99]. In each case, the condensate assembles reversibly under stress or during normal RNA metabolism [97], and dissolves once conditions normalize [100,101], precisely the defining signature of the disordome as developed in this article. A striking example is also NUPR1, a small intrinsically disordered nuclear protein that drives stress granule formation via LLPS in pancreatic cancer cells, and whose pharmacological disruption induces synthetic lethality in KRAS-driven tumors [102].

Strikingly, several of these same proteins reveal what happens when this reversibility fails. TDP-43 and FUS, whose physiological condensates are liquid and dynamic, can undergo a liquid-to-solid transition driven by disease mutations [103,104,105] or chronic stress [106,107], producing the pathological aggregates characteristic of amyotrophic lateral sclerosis and frontotemporal dementia. Tau and α-synuclein show comparable behavior in Alzheimer’s and Parkinson’s disease [108,109]. These liquid-to-solid transitions in intracellular condensates do not involve raft gangliosides; the landscape-targeting strategy discussed in Section 2 applies to the membrane-initiated, ganglioside-dependent amyloid pore pathway. The disordome logic therefore cuts both ways: the very electrostatic and multivalent interactions that generate functional, reversible order under normal conditions are also what, once dysregulated, lock the system into an aberrant, essentially irreversible structured state, reframing several major neurodegenerative diseases as pathologies of a landscape rather than of a single protein conformation.

**Table 1:** Representative IDR-driven condensates.

Protein	Disordered Region	Biological Function	LLPS Mechanism	Cellular Context	Key Reference
FUS	N-terminal SYGQ-rich low-complexity domain (LCD)	RNA-binding protein, pre-mRNA splicing, transcription regulation	Multivalent tyrosine–arginine contacts drive LLPS; π–π and cation–π interactions stabilize the dense phase	Nuclear speckles, stress granules; aberrant LLPS linked to ALS/FTD	[48]
hnRNPA1	C-terminal low-complexity prion-like domain	mRNA processing, stress granule assembly	Multivalent LCD–LCD interactions; phase separation promotes stress granule formation and can seed pathological fibrillization	Cytoplasmic stress granules; disease-associated mutations increase aggregation propensity	[94]
TDP-43	C-terminal glycine-rich low-complexity domain	RNA/DNA-binding, mRNA splicing and stability	LCD-mediated multivalent interactions drive LLPS; helix–helix contacts within LCD stabilize droplets	Nuclear and cytoplasmic condensates; pathological aggregation in ALS/FTD	[105]
Ddx4 (DDX4)	N-terminal RGG/FG-rich disordered region	Germ-cell “nuage” RNA helicase, piRNA pathway	Weakly interacting blocks of charged and aromatic residues; salt- and temperature-responsive LLPS	Perinuclear nuage granules in germ cells	[87]
G3BP1	Intrinsically disordered RGG and acidic regions flanking the NTF2-like domain	RNA-binding, core stress granule scaffold	Multivalent protein–protein and protein–RNA interactions nucleate LLPS above a concentration threshold	Cytoplasmic stress granules under oxidative, ER, or metabolic stress	[110]
MED1 (Mediator complex)	Intrinsically disordered activation domain	Transcriptional coactivator, super-enhancer function	Phase separation of coactivators concentrates transcriptional machinery at super-enhancers	Nuclear transcriptional condensates at super-enhancer loci	[111]
BRD4	Intrinsically disordered low-complexity domain	Chromatin reader, transcriptional coactivator	Activation-domain-driven LLPS; disordered domains of transcription factors phase-separate to activate transcription	Nuclear condensates at active gene loci	[111]
NUPR1 (p8)	Full-length protein (82 residues, monomeric IDP)	Stress-associated transcriptional regulator; translation control via eIF2α interaction; cell survival	NUPR1-driven LLPS forms stress granules; pharmacological disruption (ZZW-115) blocks droplet formation and induces synthetic lethality	Pancreatic cancer cells (KRAS G12D); endoplasmic reticulum and metabolic stress	[102]

## 4. Defining the Disordome: From the Unfoldome to Reversible Order

Because IDPs constitute a substantial fraction of most proteomes, it has been proposed to group them into an “unfoldome”, defined as the portion of the proteome comprising all natively unstructured or unfolded proteins [11]. Here, the term disordome is introduced to denote something more specific and more dynamical: the functional universe of intrinsically disordered proteins and regions considered not merely as a set of sequences lacking fixed structure, but as a network of molecular systems whose behavior is governed by emergent order arising from disordered ensembles. In the disordome, the central object is not a static three-dimensional fold, but a landscape (more precisely an electrostatic and interaction landscape) through which chains move and in which local, transient reductions in conformational and solvent entropy generate biologically meaningful events such as specific binding, allosteric regulation, and phase separation.

By definition, the disordome encompasses IDPs/IDRs that use their native disorder to support context-dependent structuring: folding-upon-binding, fuzzy complexes, multivalent interaction hubs, and condensates formed by liquid–liquid phase separation. What unifies these systems is that their functional “units” (binding domains, scaffolds, droplets) do not exist as fixed, pre-formed structures in the isolated state. Instead, they emerge reversibly when appropriate partners, cofactors, or physicochemical conditions reshape the underlying electrostatic and hydration landscape. From the microscopic point of view, individual atoms simply respond to local fields; at the mesoscopic level, reproducible patterns of order appear and disappear, encoding and processing biological information while remaining fully consistent with the second law of thermodynamics.

In short, the unfoldome is the subset of the proteome that is structurally unstructured, whereas the disordome is the subset of biological phenomena in which intrinsic disorder is actively exploited to generate emergent, reversible order. The disordome shifts the focus from “what structure does this sequence have?” to “what emergent structures and phases can this disordered ensemble support, under which landscape conditions, and at what thermodynamic cost?”.

To make this distinction operational and falsifiable, the disordome framework yields three concrete, testable predictions that go beyond existing descriptions of intrinsic disorder. First, one destination per ligand. Because binding-competent conformations are funneled by the specific partner’s electrostatic field rather than pre-encoded once and for all in the sequence, the same IDP engaging structurally distinct partners should adopt measurably distinct bound-state conformational ensembles. This is directly testable by comparative biophysical methods (e.g., NMR [112], HDX-MS [113], ion-mobility MS [114], or smFRET [115]) across multiple ligands of a single IDP, i.e., methods that can resolve whether a single disordered sequence populates partner-specific structural sub-ensembles rather than a single, partner-independent ensemble. Second, partner-diversity/predictability trade-off. Regions engaging a larger number of structurally distinct partners should show systematically lower AlphaFold confidence (pLDDT) and higher hallucination rates than regions with a single dominant partner or none, independent of overall disorder propensity. This is retrospectively testable using existing AlphaFold outputs and curated interactome data, and would provide a quantitative, sequence-level signature of the disordome logic [116]. Third, asymmetric entropic partitioning. The entropic cost of IDP–partner complex formation should be measurably redistributed toward the partner and solvent, relative to comparable folded-protein interfaces of similar buried surface area, and should be quantifiable by isothermal titration calorimetry [117] combined with NMR-based estimates of conformational entropy [28,118,119] and with computational decomposition of conformational versus solvent contributions [120]. These three predictions are specific, falsifiable consequences of treating the electrostatic landscape, rather than the disordered sequence alone, as the primary unit of functional organization.

The disordome framework does not replace the established concepts of fuzzy complexes, fly-casting, and high-affinity disordered complexes. It situates them within a single vocabulary. Fuzzy complexes [121] are the case in which functionally relevant disorder is retained even in the bound state, whether as alternative bound conformations, flexible clamps between binding sites, disordered flanking regions, or fully disordered “random” complexes. In disordome terms, the bound state is then a landscape that remains partly open rather than a collapsed structure. Fly-casting [122] is the kinetic counterpart of the encounter phase described above. A partially unstructured chain has a larger capture radius than its folded counterpart, binds weakly at a distance, and folds as it approaches its target. Electrostatic steering, which biases the orientation and approach of the chain, is one physical contribution to this larger capture radius. We stress, however, that fly-casting was originally formulated in terms of the folding funnel and not of electrostatics alone, and that it mainly accelerates association, whereas specificity arises from the short-range contacts discussed in Section 2. High-affinity disordered complexes represent the limiting case. The histone H1–prothymosin-α complex associates with picomolar affinity yet remains highly disordered and dynamic, an interaction explained largely by the large opposite net charge of the two partners and not by defined binding sites [123]. Here, funneling by a single, dominant partner is so effective that it confers very high affinity without eliminating the ensemble character of the chain. Fuzzy complexes, fly-casting, and ultrahigh-affinity disordered complexes can thus be read as three positions along one continuum, from the partial retention of disorder after binding, to kinetic acceleration of the encounter, to maximal affinity with almost complete retention of disorder. The three predictions above apply to all three.

## 5. Historical Precursors and the Physical Basis of Conformational Freedom: Bernal, Klotz, and the Limits of AlphaFold

Long before the disordome, or even the unfoldome, was named, some of the most acute physical chemists of the mid-twentieth century had already glimpsed its logic. In 1962, in his chapter “The Structure of Molecules” for Comprehensive Biochemistry (edited by Marcel Florkin and Elmer Stotz), the crystallographer J. D. Bernal wrote that the physical properties of proteins “which cannot as yet be crystallized”, particularly their possibility of extension and “a certain degree of rubber-like elasticity”, indicate the presence of long molecules “which can be coiled in different ways” [17]. This sentence anticipates, with startling precision, the modern description of an IDP as a dynamic conformational ensemble rather than a single fold. Bernal’s operational criterion (in fact resistance to crystallization) is, in essence, the very criterion that would later define IDPs by their inability to yield a unique structure by X-ray diffraction. His recourse to rubber elasticity already imports, four decades before Wright and Dyson’s 1999 review consecrated the field [2], the physics of flexible polymer chains as the correct model for a class of proteins whose function does not derive from a fixed architecture.

**Figure 5:**
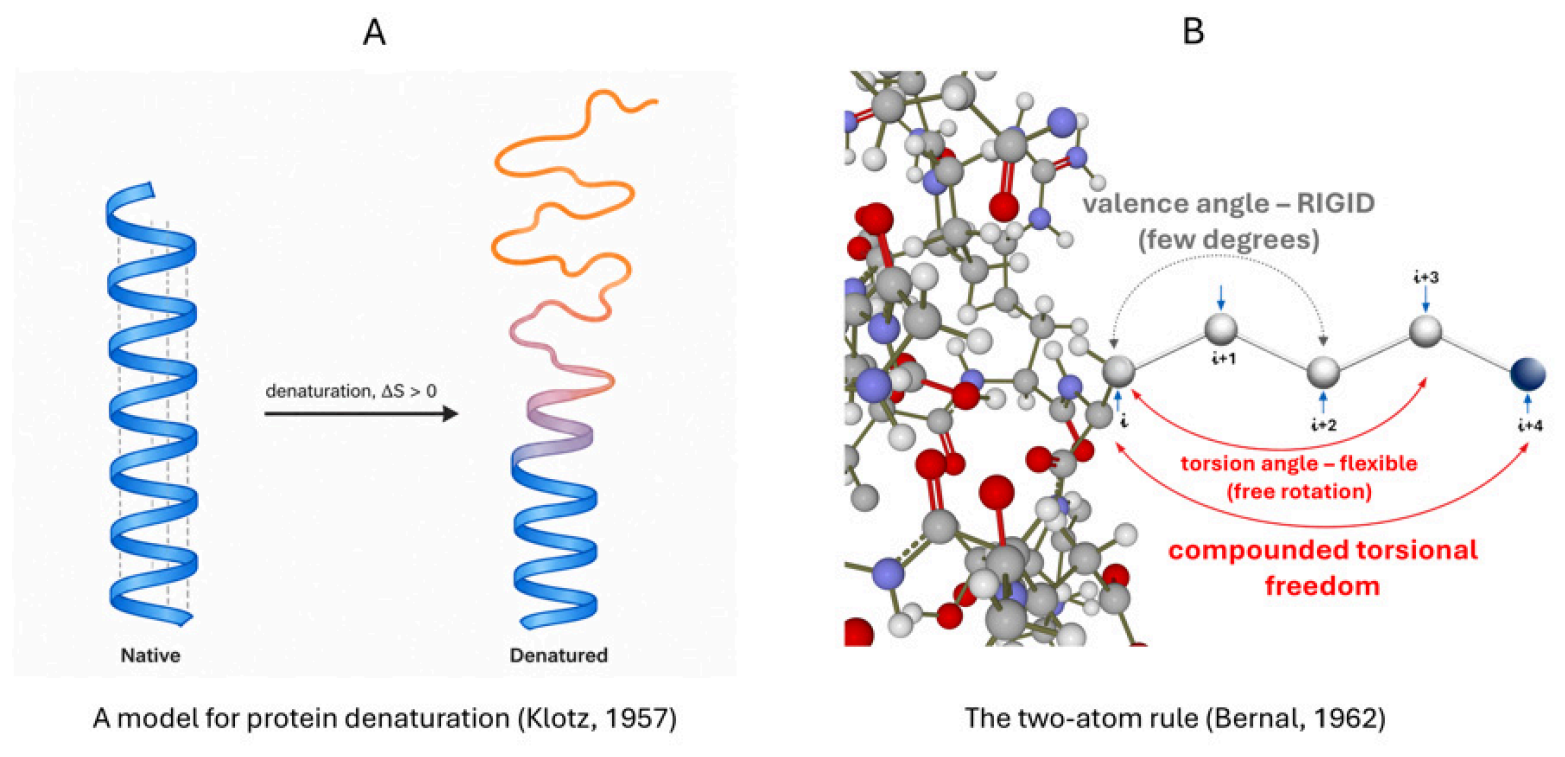
Two mid-twentieth-century intuitions of protein flexibility, reconsidered in light of AlphaFold’s limitations. (**A**) Klotz’s 1957 model of protein denaturation [16]. A regular, hydrogen-bonded α-helix (native state, blue) progressively unwinds into an increasingly irregular coil before ending in a fully disordered, randomly fluctuating segment (denatured state, orange). Klotz used this transition to argue that the large positive entropy change (ΔS > 0) associated with denaturation reflects the fact that the protein becomes “disoriented”, a term that, forty years before the formal recognition of intrinsic disorder, already captured the physical essence of a disordered conformational ensemble. (**B**) Bernal’s 1962 two-atom rule [17] illustrated on a lysine side chain. The side chain is taken from the V3 loop of HIV-1 gp120 (PDB 1CE4); atoms are numbered from Cβ (i) to Nζ (i + 4). Bond lengths and the valence angle (grey dotted arc, i to i + 2) fix the relative positions of atoms up to two bonds apart, which remain rigid to within a few degrees. Conformational freedom appears from the 1,4 relationship onward (red arc, i to i + 3, e.g., Cβ to Cε), through torsion about single, rotatable bonds, and compounds along the chain (large red arc, i to i + 4) up to the terminal ε-amino nitrogen (Nζ, blue), which carries the positive charge responsible for lysine’s electrostatic contacts.

This rubber analogy captures a precise thermodynamic mechanism, independently elaborated by the physical chemist Irving Klotz in his 1957 monograph Some Principles of Energetics in Biochemical Reactions [16]. Klotz invokes the same image—a stretched rubber band that spontaneously contracts back to its disordered state with ΔE ≈ 0—to introduce entropy as the true driving force of spontaneous processes that energy minimization alone cannot explain. His accompanying figure of the “configurations of macromolecules in rubber”, contrasting an ordered, extended chain “at rest” with a relaxed, multiply-coiled state, is, avant la lettre, a portrait of a conformational ensemble. Elsewhere in the same book, describing protein denaturation, Klotz writes that the large associated entropy increase reflects the fact that “the protein molecule becomes disoriented”, a term poised, both semantically and conceptually, exactly halfway between “denatured” (the 1957 vocabulary) and “intrinsically disordered” (the 1999 vocabulary). His accompanying diagram of a native, fully helical protein next to a “denatured” one, showing a partial helix flanked by disordered coil regions, prefigures the now-standard cartoon of an IDR-containing, mixed-architecture protein (Figure 5A). What Klotz lacked was not the physics, which was essentially correct, but the biological reframing that would, forty years later, recognize this “disoriented” state as a native, functional condition rather than a pathological deviation. A corollary, still insufficiently appreciated, is that the term “misfolded proteins”, often used to designate the cause of diseases involving IDPs, is hardly appropriate for proteins that have no native fold to begin with [124], and we suggest that it be replaced by more accurate descriptions such as pathological conformational conversion [125].

Bernal went further still. In the same 1962 chapter [17], in a passage on “Rigid and Flexible Molecules”, he located the physical root of this conformational freedom in the range of electronic constraint itself: “the reason for the existence of alternatives, or wide ranges of possible conformation, is the limited degree to which the electronic nature of the bonds fixes the mutual position of atoms more than two atoms apart.”. This statement, here termed the two-atom rule, can be stated more precisely. Bond lengths and bond angles fix the relative positions of atoms up to two bonds apart (1,2 and 1,3 pairs); conformational freedom appears from the 1,4 relationship onward, through torsions about bonds (Figure 5B). Single bonds allow rotation, whereas the partial double-bond character of the peptide bond, due to resonance, enforces near-planarity of the amide group and removes one torsional degree of freedom per residue (the ω dihedral); likewise, electron delocalization keeps aromatic rings planar. The conformational freedom of a polypeptide chain therefore resides essentially in the backbone torsions φ and ψ, which the Ramachandran diagram, published the following year, would formalize [126]. The rule is thus a statement about local covalent geometry, valid for single bonds and modified for partial double bonds, not a claim that electronic influence decays smoothly with distance. Successive residues therefore remain geometrically under-constrained relative to one another unless an additional network of longer-range, non-covalent interactions locks them into place.

This mechanism can now be reframed (as a working hypothesis rather than an established result) in terms of what may be called a preferential folding pathway. In a globular protein, the geometry of nonpolar side chains does not merely tolerate packing in the apolar core; it actively constrains it, since hydrophobic residues cluster according to strict rules of geometric complementarity and van der Waals cooperativity rather than at random [127,128]. From a thermodynamic point of view, it is important to mention that the entropy gain of water that accompanies folding and collapse is mainly translational because the solvent-excluded volume effect is the main actor; the degrees of freedom that play a role are not the rotational ones (those associated with water–water H-bonds) [129,130]. This solvent-excluded-volume effect can drive the collapse of polypeptides devoid of nonpolar side chains, such as polyglycine [131], so nonpolar residues are not required for collapse as such; they are essential, rather, for the attainment of a unique folded structure. In the framework developed here, the translational entropy of water thus supplies a generic driving force [132], whereas the packing of nonpolar side chains supplies the specificity that defines a preferential pathway.

This packing progressively overrides the local under-determination described by the two-atom rule: as successive nonpolar side chains lock into a cooperative network, the astronomically large space of initially accessible conformations collapses onto essentially one reproducible route to one dominant structure, that is, the single preferential pathway that Anfinsen’s paradigm takes as the default behavior of proteins [1]. IDPs and IDRs lack the amino-acid composition needed to generate this packing force: their side chains are predominantly polar or charged, each with greater affinity for surrounding water than for a neighboring residue, so the two-atom rule’s local under-determination is never resolved into a stable long-range network. In the absence of a partner, there is therefore no preferential pathway at all, only the broad, entropically dominated ensemble already described. This changes, however, the moment a ligand engages the chain. A ligand’s electrostatic field can perform, for an IDP, the same funneling role that van der Waals packing performs for a globular protein: it selectively stabilizes a subset of local, non-covalent contacts (electrostatic, hydrogen-bonding, OH–π, or dispersion in nature) that channel the ensemble toward one binding-competent conformation. Because this funneling is ligand-specific rather than encoded once and for all in the sequence, an IDP can, in principle, possess as many preferential pathways as it has functionally relevant partners, one path per ligand, each carved out anew from the same disordered ensemble. A globular protein, in contrast, funnels through many concurrent microscopic trajectories toward a single native destination, fixed by the sequence alone and independent of which partner it may subsequently engage: multiplicity of routes coexists here with a unicity of outcome. For an IDP, this relationship is inverted. In the absence of a partner, the chain’s many accessible trajectories lead to different conformations constituting the disordered ensemble, with no single destination privileged by the sequence. Once a ligand engages the chain, however, its field does not simply select among pre-existing routes toward a pre-fixed destination; it may determine the destination itself. Structurally distinct partners can therefore carve out structurally distinct preferential funnels from the same underlying disordered ensemble, each converging on its own binding-competent conformation, one destination per ligand rather than one route per protein.

This multiplicity of ligand-conditioned pathways remains, for now, a conceptual extrapolation from the two-atom rule rather than a systematically demonstrated property: it is documented in specific cases such as α-synuclein’s distinct engagement of GM3 versus other partners [19,68], but has not yet been tested comparatively across multiple ligands of a single IDP. We offer it here as a testable hypothesis that would, if confirmed, help explain why an IDP itself is an elusive pharmacological target, and why targeting the landscape that dictates which pathway is selected may be more tractable than targeting the disordered protein in isolation [19].

The two-atom rule provides the physical background that may explain why AlphaFold and related deep-learning structure predictors systematically fail on IDPs and IDRs [133,134,135]. Torsional freedom is of course shared by folded and disordered proteins; what distinguishes them is whether a conserved network of non-covalent contacts resolves this freedom into a unique structure, which is what AlphaFold exploits and what IDPs lack. A further limitation is the scarcity of experimentally determined ensembles on which a predictor could be trained [136]. AlphaFold’s architecture is built to exploit exactly the kind of long-range geometric determinism that Bernal’s rule identifies as characteristic of folded domains: it infers a single, static three-dimensional arrangement from co-evolutionary correlations across a multiple sequence alignment [137], correlations that exist precisely because a locked network of long-range non-covalent contacts constrains residues far apart in sequence to co-vary in a folded structure [138]. For IDPs and IDRs, no such co-evolutionary signal exists because there is no persistent long-range network to select for: sequence variability is high [139], and the very electronic under-determination that Bernal described translates, at the level of a multiple sequence alignment, into the absence of the correlated substitutions the network requires [140,141].

Empirically, this is exactly what is observed. Regions of very low AlphaFold confidence (pLDDT below 50) correlate strongly with intrinsically disordered regions [140], and AlphaFold nonetheless outputs a single static conformation (typically an extended, “floating” loop) for segments that in reality sample a broad ensemble [142]. For canonical IDPs such as α-synuclein, Tau, or the p53 transactivation domain, AlphaFold returns fragmented, low-confidence, essentially uninformative models [143], even though these proteins are functionally essential, and as this article argues, achieve their function precisely through the disordered ensemble that AlphaFold cannot represent [144]. A recent preprint analyzing AlphaFold3 reports outright “hallucinations” in disordered regions—confidently predicted local order where none exists, or vice versa—affecting roughly one-fifth to one-third of residues within disordered proteins [145]. These data suggest that the problem is not simply insufficient training data [146] but a mismatch of paradigm: a single-structure predictor confronting a fundamentally ensemble-based class of molecules [135].

This claim now rests on more than the architectural argument developed above. The CASP16 Ensemble Prediction experiment, the most recent (December 2024) blind, community-wide assessment of exactly the “emerging ensemble generators” that might be expected to resolve this problem, found that across ten targets requiring multi-state or flexible modeling, no prediction group produced a high-accuracy model except for the single target for which a pre-existing apo structural template was available [78]. Canonical intrinsically disordered protein ensembles could not even be included in the blind target set, because no scoring protocol against a single ground-truth structure exists for them. This stands in stark contrast to the same CASP16 round’s assessment of ordinary folded targets, which concluded that single-domain fold prediction is “nearly solved” [147]. The gap between these two outcomes, within the same blind competition and the same generation of methods, is consistent with the asymmetry the two-atom rule predicts.

Bernal’s two-atom rule thus offers the disordome paradigm a physical framework for a fact that would otherwise remain purely descriptive. The failure of structure prediction for flexible and multi-state systems, including IDPs, which has persisted through the most recent community-wide blind assessment [78] is not merely a temporary training-data deficiency; it may also reflect the same physical principle that makes intrinsic disorder possible in the first place. Predicting “the” structure of an IDP is, in this sense, a mismatch of paradigm [135], akin to asking for “the” shape of a piece of rubber at rest. Emerging generative models trained on simulation data can produce IDP ensembles directly from sequence [136,148,149]; within the limits of the force fields used for training; they do so by abandoning the single-structure paradigm, consistent with the argument above. The partner-conditioned layer of the landscape, namely how the field of a given partner reshapes the statistical weight of pre-organized microstates, which residues steer the encounter, and under which conditions a transient, reversible order emerges, has only begun to be addressed, and is what the disordome paradigm invites the field to pursue. Six decades after Bernal and Klotz reached for rubber to describe what a crystallographer’s eye could not otherwise categorize, the same image remains the most physically honest one available for the disordome: not architecture, but elasticity.

Any historical account of the intrinsically disordered protein concept is necessarily a matter of selection. Uversky and Kulkarni’s recent and thorough chronology of the field’s discovery traces a rich set of twentieth-century antecedents [150], from the 1939–1940 immunochemical speculations of Landsteiner and Pauling on antibody flexibility, through the biophysical characterization of casein and phosvitin in the 1950s, to the mid-1960s optical-rotation classifications, and ultimately, the field-defining 1999 review by Wright and Dyson. Given the sheer breadth of literature such a chronology must survey, it is unsurprising that it does not mention either Bernal’s 1962 remarks on the rubber-like elasticity of non-crystallizable proteins or Klotz’s 1957 discussion of protein “disorientation”. Every chronology reflects the corpus consulted and the disciplinary vantage point of its author, and none can be exhaustive. The precursors discussed below should therefore be read as complementing, rather than correcting, Uversky and Kulkarni’s chronology, adding two additional, and strikingly explicit, threads to an already rich prehistory of the disordome.

## 6. Beyond Classical Force Fields: Quantum-Chemical Contributions to the Disordome

All chemical interactions are, at some level, quantum-mechanical in origin, including the Coulombic electrostatics already central to the disordome framework developed above. Our point here is narrower: several specific interactions relevant to disordered-protein behavior—dispersion beyond simple Lennard–Jones approximations, anisotropic OH–π and lone pair–π contacts, and proton/hydrogen tunneling—are not adequately captured by the classical, non-polarizable force fields and point-charge electrostatic models that dominate current computational and conceptual treatments of IDPs, and require explicit quantum-chemical parameterization to be described accurately [151]. We argue that this gap is comparatively underrepresented in the IDP literature relative to the five classical parameters (water, time, space, entropy, electrostatic potential) discussed above, and we present the following considerations as an exploratory outlook rather than an established body of evidence specific to disordered systems.

A concrete starting point comes from glycosphingolipid recognition by α-synuclein. Binding of α-synuclein to ganglioside-containing membranes, such as GM3 or GM1-rich rafts, depends critically on Tyr39 [67] and on the formation of a specific glycosphingolipid-binding motif at the membrane interface [68]. Detailed studies indicate that the driving force for insertion involves a transient OH–π hydrogen bond between the ganglioside and the aromatic ring of Tyr39, which nucleates membrane insertion and progressive structuring of the ganglioside-binding domain [19]. This OH–π contact, while often treated as a “geometric” motif, is a genuinely quantum object: its stability and directionality depend on the anisotropic distribution of π-electron density, on polarization, and on dispersion contributions [152]. More generally, lone pair–π and OH–π interactions between water oxygens or polar headgroups and aromatic residues are now recognized as widespread in protein structures [153] and require quantum-chemical treatment to be described accurately [154]. In the disordome, such interactions are not rare exceptions; they are repeatedly formed and broken as disordered chains explore their conformational landscape and encounter membranes, partners, and solvents.

Beyond specific OH–π contacts, quantum mechanics enters the disordome through the myriad weak interactions that collectively stabilize transient motifs and encounter complexes. Dispersion forces and van der Waals interactions, intrinsically quantum in nature, are particularly important in the diffuse, “fluffy” environments characteristic of IDRs, where no single set of close-packed contacts dominates [155,156]. In a disordered ensemble, the effective “glue” that brings side chains together into short-lived helices, turns, or fuzzy interfaces arises from the sum of many individually weak interactions whose energies and anisotropies are determined by an underlying quantum Hamiltonian. In this sense, quantum dispersion can be viewed as one of the microscopic currencies with which the disordome pays for local emergent order.

Quantum tunneling [157] offers a second class of phenomena that may intersect the disordome in nontrivial ways. Proton and hydrogen tunneling are well-established in enzyme catalysis, where the rate of barrier crossing depends sensitively on donor–acceptor distances, local electric fields, and the organization of the hydrogen-bond network [158]. In structured enzymes, conformational dynamics and electrostatics are known to tune tunneling probabilities by modulating these parameters over time [158,159,160]. Intrinsically disordered segments have so far received little attention in this context, yet they are often juxtaposed to catalytic cores or regulatory domains. Their large-amplitude fluctuations and capacity to reshape the local electrostatic and hydration environment suggest that IDRs could act as dynamic modulators of proton- or electron-tunneling events, even if the tunneling itself occurs within a more structured region. Within the disordome paradigm, this would represent another form of emergent function: quantum events that are not carried by the disordered chain, but whose rates and selectivities are encoded in the disordered electrostatic landscape that the chain generates.

Water, finally, provides a natural bridge between intrinsic disorder and quantum biology. In structured proteins, there is growing interest in how ordered hydration shells participate in electron transfer, proton conduction, and the stabilization of particular conformations via networks of hydrogen bonds with quantum-sensitive properties [161]. IDPs sit at the opposite extreme, as already noted above. Yet the same types of interactions (hydrogen bonds, lone pair–π contacts with aromatic residues, cooperative polarization within clusters of water) are present, only more dynamic and more broadly sampled in time. The disordome thus extends into a regime where quantum-informed water interactions are not organized around a rigid scaffold, but around a fluid, constantly reshaping polymer, opening the possibility that quantum contributions to noncovalent interactions and proton/electron transfer are averaged in a way that is functionally exploitable.

**Figure 6:**
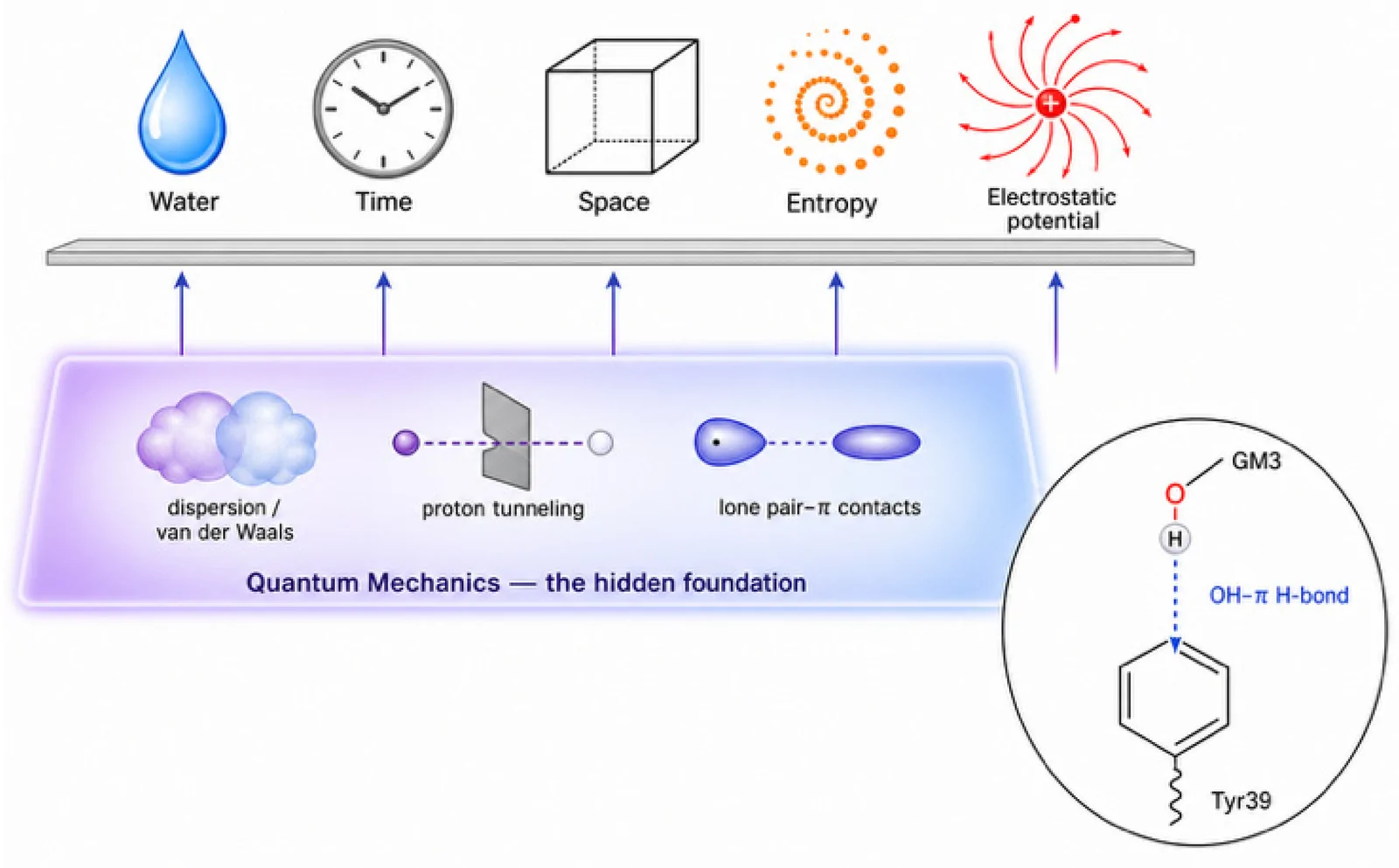
Interactions beyond classical force fields as an underrepresented layer of the disordome. Most discussions of intrinsically disordered proteins rely, explicitly or implicitly, on five recognized physical pillars, i.e., water, time, space, entropy, and electrostatic potential (top row), each associated with a well-documented aspect of IDP behavior, from hydration dynamics to electrostatic steering. This figure represents these five pillars as resting on a foundation of specific interactions (bottom slab) that are inadequately captured by the classical, non-polarizable force fields and point-charge electrostatic models dominating current IDP simulation and conceptual treatment, and that require explicit quantum-chemical parameterization to be described accurately: dispersion beyond simple Lennard–Jones approximations, which provides the diffuse, non-specific “glue” stabilizing transient motifs in the absence of close-packed contacts; proton and hydrogen tunneling, which can modulate reaction rates at catalytic sites juxtaposed to flexible segments; and anisotropic lone pair–π contacts between water and aromatic residues, which shape local hydration. The inset (lower right) illustrates a concrete instance of this layer: the OH–π hydrogen bond thought to nucleate α-synuclein’s insertion into ganglioside-containing membranes. This single, transient contact exemplifies how an interaction poorly described by classical force fields, blurred by thermal noise and repeatedly formed and broken as the disordered chain samples its conformational ensemble, can nucleate and steer the emergence of macroscopic, functionally meaningful order, linking this underrepresented layer to the five classical pillars shown above.

In summary, quantum mechanics is the microscopic layer beneath the five better-recognized physical parameters that already structure IDP research, i.e., water, time, space, entropy, and electrostatic potential (Figure 6) [58]. What is currently missing is not the presence of quantum effects, but an explicit language to relate them to emergent functional phenomena in intrinsically disordered systems. Recognizing this underrepresentation reframes the disordome as a natural arena for “What is life?”-type questions [58]: How do quantum-defined interactions, blurred by thermal noise and explored over vast conformational ensembles, give rise to reproducible local entropic emergences that encode biological function? Addressing this will require new bridges between quantum chemistry, statistical mechanics, and IDP biophysics, and may reveal that some of the most subtle aspects of life’s organization are already hiding in plain sight within the fluctuating world of disordered proteins.

Taken together, these considerations suggest that the disordome is a particularly transparent arena in which to connect quantum mechanics to the fundamental question “What is life?” [58]. Life can be viewed as a process based on molecular interactions that organize controlled local entropic emergences, regulated by the mutual influences that biomolecular partners exert on the electrostatic and hydration landscape. In intrinsically disordered systems, these emergences (including transient binding domains, fuzzy interfaces, and condensates) are directly shaped by quantum-defined interactions that are continuously explored and averaged over broad conformational ensembles. The disordome paradigm thus makes explicit that biological function is not only compatible with the second law of thermodynamics, but also rooted in quantum mechanics: intrinsic disorder provides the fluctuating canvas on which quantum-level interactions are sculpted into reproducible patterns of order that encode, process, and transmit biological information. As stated at the outset, these connections are proposed as hypotheses to be tested rather than as established mechanisms; their value lies in the questions they generate for future quantum-chemical and biophysical work, not in a claim of proof already delivered.

## 7. Conclusions and Perspectives

Beyond its immediate explanatory value, the disordome paradigm carries implications that extend well beyond the biophysics of individual proteins. At the level of fundamental biology, recognizing that function can be encoded in landscapes rather than in fixed folds reopens the question of the origin of life in a new light: if reproducible order can emerge, reversibly and at low entropic cost, from flexible, poorly-constrained chains obeying nothing more than local electrostatic and hydration rules, then the classical requirement of a pre-existing, highly specified, folded catalyst as a precondition for early biochemistry becomes far less stringent—primitive peptides, RNA-protein assemblies, and coacervate-like compartments (the very objects Oparin and Dyson already intuited) may have exploited disorder, not structure, as their first functional currency. At the level of cellular function, the disordome reframes signaling hubs, transcription factor networks, and biomolecular condensates not as accidental byproducts of “unfolded” sequences but as a genuine regulatory layer in which promiscuity, multivalency, and conditional folding are the very source of combinatorial complexity that a rigid, one-structure–one-function proteome could never achieve—a layer whose systematic mapping (a true “disordome-wide” atlas of landscapes, analogous to the genome or the interactome) remains largely to be built. At the level of medicine, the disordome paradigm suggests a complementary strategy to the direct targeting of disordered proteins, which has proven difficult and has been approached via several routes, including designed binders and small molecules that disrupt IDP-driven condensates [77,79,102]. The strategy proposed here is to occupy the electrostatic landscape that recruits and structures the IDP, rather than the IDP itself. This principle, developed in mechanistic and pharmacological detail elsewhere [19], is illustrated by the adaptive peptide AmyP53, which competes with amyloid proteins for raft gangliosides and has been well-tolerated in short-term preclinical studies [69]. Whether the landscape is a more tractable target than the disordered protein remains to be tested, notably for long-term safety, off-target effects on physiological ganglioside functions, and efficacy in humans. This strategy, termed “target the target, not the arrow”, could in principle extend beyond neurodegeneration to other charged biological surfaces (DNA, glycosaminoglycans, other anionic membrane domains) that recruit and structure a disordered partner.

## Data Availability

No new data were created or analyzed in this study. Data sharing is not applicable to this article.

## Abbreviations

CBD	Cholesterol binding domain
GBD	Ganglioside binding domain
HDX-MS	Hydrogen/deuterium eXchange mass spectrometry
IDPs	Intrinsically disordered proteins
IDRs	Intrinsically disordered regions
LCD	Low complexity domains
LLPS	Liquid–liquid phase separation
MS	Mass spectrometry
NMR	Nuclear magnetic resonance
pLDDT	Predicted local distance difference test
RF	RoseTTAFold
SGs	Stress granules
smFRET	Single-molecule fluorescence resonance energy transfer
